# Intratumoral CD3+ T-Lymphocytes Immunoexpression and Its Association with c-Kit, Angiogenesis, and Overall Survival in Malignant Canine Mammary Tumors

**DOI:** 10.1155/2015/920409

**Published:** 2015-08-05

**Authors:** Maria Isabel Carvalho, Isabel Pires, Marlene Dias, Justina Prada, Hugo Gregório, Luis Lobo, Felisbina Queiroga

**Affiliations:** ^1^CECAV, University of Trás-os-Montes and Alto Douro, 5001-801 Vila Real, Portugal; ^2^Department of Veterinary Sciences, University of Trás-os-Montes and Alto Douro, 5001-801 Vila Real, Portugal; ^3^School of Life Sciences and the Environment, University of Trás-os-Montes and Alto Douro, 5001-801 Vila Real, Portugal; ^4^Centro Hospitalar Veterinário, Rua Manuel Pinto de Azevedo 118, 4100-320 Porto, Portugal; ^5^Hospital Veterinário do Porto, Travessa Silva Porto 174, 4250-475 Porto, Portugal; ^6^Faculdade de Medicina Veterinária, Universidade Lusófona de Humanidades e Tecnologias, Campo Grande 376, 1749-024 Lisboa, Portugal; ^7^Center for the Study of Animal Sciences, CECA-ICETA, University of Porto, 4051-401 Porto, Portugal; ^8^Center for Research and Technology of Agro-Environment and Biological Sciences (CITAB), University of Trás-os-Montes and Alto Douro, 5001-801 Vila Real, Portugal

## Abstract

In this study 80 malignant CMT were submitted to immunohistochemical detection of CD3, c-kit, VEGF, and CD31, together with clinicopathological parameters of tumor aggressiveness. CD3+ T-cells and c-kit overexpression revealed a positive correlation with VEGF (*r* = 0.503,* P* < 0.0001;* r* = 0.284,* P* = 0.023 for CD3 and c-kit, resp.) and CD31 (*r* = 0.654,* P* < 0.0001;* r* = 0.365,* P* = 0.003 for CD3 and c-kit, resp.). A significant association (*P* = 0.039) and a positive correlation (*r* = 0.263,* P* = 0.039) between CD3 and c-kit were also observed. High CD3/VEGF, c-kit/VEGF, and CD3/c-kit tumors were associated with elevated grade of malignancy (*P* < 0.0001 for all groups), presence of intravascular emboli (*P* < 0.0001 for CD3/VEGF and CD3/c-kit; *P* = 0.002 for c-kit/VEGF), and presence of lymph node metastasis (*P* < 0.0001 for all groups). Tumors with high CD3/VEGF (*P* = 0.006), c-kit/VEGF (*P* < 0.0001), and CD3/c-kit (*P* = 0.002) were associated with poor prognosis. Interestingly high c-kit/VEGF tumors retained their significance by multivariate analysis arising as independent prognostic factor.

## 1. Introduction

Tumor-associated inflammatory response is a highly coordinated event involving several key players and have the effect of enhancing mammary tumorigenesis, helping emerging neoplasias to acquire hallmark capabilities [1, 2]. Chronic inflammation contributes to activation of important facilitating programs by providing active molecules to the tumor microenvironment, including growth and survival factors; proangiogenic factors as VEGF; and extracellular matrix-remodeling enzymes that allow angiogenesis, invasion, and metastasis [3–5]. Additionally, inflammatory cells can contribute to mutagenic transformation of cancer cells, accelerating their genetic evolution toward states of higher malignancy [6, 7]. However, how the chronic inflammation in the mammary tumor microenvironment is coordinated by inflammatory cells themselves is still incompletely understood.

Recent studies, in human breast cancer [8, 9] and canine mammary tumors (CMT) [10–12], highlight T-lymphocytes as an important regulator of inflammation and their accumulation in tumor sites has also been well documented [8, 10]. T-cells migration to tumor site and the following activation may be the essential requirement for their local promoting effect [2, 13, 14]. Nevertheless, how T-lymphocytes are recruited into the tumor site and whether they can remodel the tumor microenvironment are key questions that remain unclear. In several human tumors, including breast cancer, the c-kit signaling has been described as being implicated in differentiation and migration of T-cells in tumor sites [15, 16]. The deregulation of c-kit induces the activation of several signaling pathways that can result in chronic inflammation with immune balance from activation to tolerance [15, 17, 18] which may be a deleterious immune condition in a variety of diseases, including cancer, and may be implicated in mammary tumorigenesis.

Nevertheless all the evidence, overexpression of c-kit, still represents a highly controversial subject in breast cancer. Several studies propose that the loss of c-kit expression has been associated with tumor progress, whereas other reports indicate overexpression of c-kit related to increase of angiogenesis and tumor development [19–24].

In CMT few studies have examined the expression of c-kit suggesting that c-kit mutation and activation may be involved in the pathogenesis of these tumors [25–27]. However, to our knowledge, there are no studies in human breast cancer or in CMT that focus on relationship between T-lymphocytes, c-kit expression, and tumoral angiogenesis and aggressiveness. Present study aims to explore the possible common signaling/regulatory pathways between c-kit and T-lymphocyte responses in CMT which may potentiate applications in immunological therapeutic strategies and provide new insights into the role of c-kit in inflammation, immunosuppression, and tumor progression.

## 2. Materials and Methods

### 2.1. Mammary Tumors and Clinicopathological Variables

This study included 80 malignant CMT excised, with curative intent, from dogs received for diagnosis and treatment. Samples were fixed in 10% formalin, processed using an automatic tissue microprocessor, and paraffin-embedded. Paraffin wax blocks were cut into 2-3 mm sections using a microtome, mounted on glass slides, and stained with hematoxylin-eosin for diagnostic purposes, according to the WHO criteria for CMT [28]. Tumors were graded in accordance with the method proposed by Goldschmidt and colleagues [29]. The following clinicopathological parameters were evaluated in each sample: tumor size (T1 < 3 cm; T2 ≥ 3 and <5 cm; T3 ≥ 5 cm), skin ulceration, tumor necrosis, mitotic index, nuclear grade, differentiation grade, histological grade of malignancy, neoplastic intravascular emboli, and regional lymph node involvement.

### 2.2. Immunohistochemical Technique

Immunoexpression of CD3, c-kit, CD31, and VEGF was carried out using the streptavidin-biotin-peroxidase complex method, with a commercial detection system (Ultra Vision Detection System; Lab Vision Corporation, Fremont, California, USA) following the manufacturer's instructions. All slides were subjected to microwave antigen retrieval before immunolabelling, with a citrate buffer, for 3 × 5 min at 750 W.

As the primary specific antibody we used CD3 (polyclonal antibody; Dako, Glostrup, Denmark; 1 : 50 dilution), during 2 hours at room temperature, c-kit (a polyclonal rabbit anti-human c-kit protein; Dako, Carpinteria, California, USA; 1 : 100 dilution), CD31 (Clone JC70A, Dako, Glostrup, Denmark; 1 : 20 dilution), and VEGF (Clone JH121, Thermo Scientific, Waltham, MA USA; 1 : 100 dilution), in overnight incubation at 4°C. Immunoreaction was visualized by slide incubation with 0.05% 3,3′-diaminobenzidine tetrahydrochloride (DAB) with 0.01% hydrogen peroxide (H_2_O_2_) as the final substrate, for 10 min. After a final washing in distilled water, the sections were counterstained with hematoxylin, dehydrated, cleared, and mounted with Entellan resin (Merck KGaA, Darmstadt, Germany). As a positive control, we used sections of canine lymph nodes for CD3; sections of cutaneous grade III mast cell tumors with known positivity to c-kit; dog angiosarcoma for CD31; and liver section for VEGF. As a negative control, the primary antibody was replaced by PBS.

### 2.3. Evaluation of Immunoreactivity

The assessment of CD3, CD31, and VEGF immunolabelling was based on methods previously used in CMT by our team [10, 30–32].

The c-kit immunoreactivity was evaluated in accordance with parameters already described in canine cutaneous melanocytic tumors [33] and in CMT [25] by determining the percentage of positively labeled cells and the labeling intensity. The percentage of positively labeled cells (extension) was scored as negative (0%), focal (1–19%), intermediate (20–49%), or diffuse (>50%). The labeling intensity was scored as negative (–), weak (+), moderate (++), or strong (+++). Subsequently, the two scores were combined (sum) and a final score was obtained: Low immunoreactivity (score values ≤ 4) and high immunoreactivity (score values 5-6). The labeling location was recorded as cytoplasmic, membranous, or both.

### 2.4. Clinical Follow-Up

Dogs were clinically examined by veterinarians every 90–120 days after surgical treatment for a minimum of 730 days. Follow-up included a radiological evaluation of the thorax and an abdominal ultrasound. For the animals that died within the 730-day period, overall survival (OS) was considered the number of days between surgery and death, whilst, for the dogs who survived > 730 days, the OS was the number of days from surgery to the last clinical examination. Follow-up was carried out for a mean period of 472 days (minimum 37 and maximum 730 days).

### 2.5. Statistical Analysis

The Chi-square test was used to study the categorical variables. Analysis of variance (ANOVA) was used for analyzing continuous variables. Pearson's correlation test was performed in order to verify the presence of correlation between values of CD3, c-kit, VEGF, and CD31. Influence on survival was established using the log rank test and cases were grouped in low or high according to expression. Cox proportional hazards model for multivariate analysis was also implemented. Analyses were performed using the statistical software SPSS (Statistical Package for the Social Sciences) version 19.0 and a conventional 5% level was used to define statistical significance.

## 3. Results

The present study comprised 38 tubulopapillary carcinomas, 14 complex carcinomas, 12 solid carcinomas, 3 anaplastic carcinomas, and 13 carcinosarcomas. Twenty malignant tumors were grade I, 20 grade II, and 40 grade III. Twenty-five tumors (31.25%) had neoplastic intravascular emboli and 31 cases (38.75%) had lymph node metastases.

### 3.1. Expression of CD3+ T-Lymphocytes, c-Kit, VEGF, and CD31 in CMT

**Figure 1:**
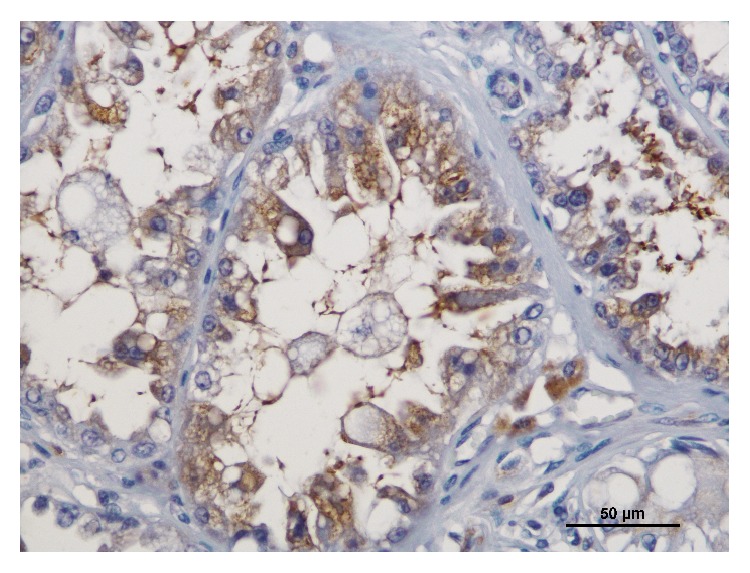
Immunoreactivity for c-kit in tubulopapillary carcinoma; note the simultaneous cytoplasmic and membranous staining; bar = 50 *μ*m.

**Figure 2:**
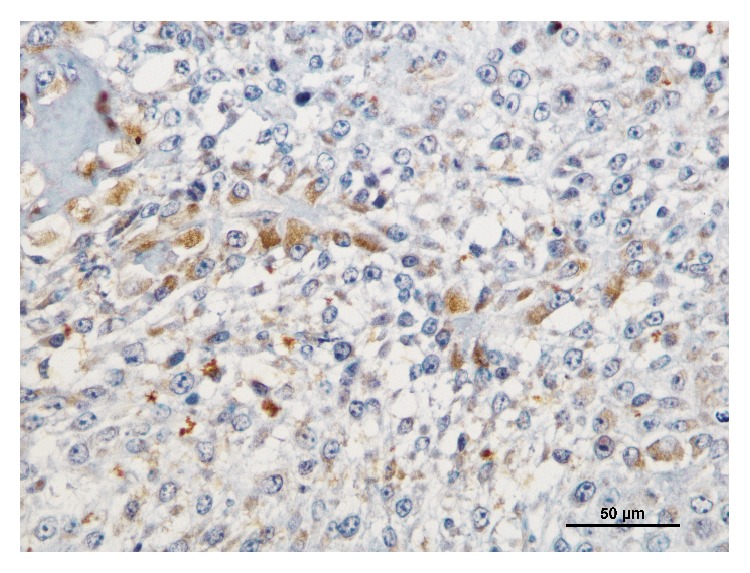
Immunoreactivity for c-kit in carcinosarcoma; bar = 50 *μ*m.

The CD3 immunostaining was observed in the cytoplasm or/and in the cytoplasmatic membrane of T-lymphocytes and the diffuse inflammation was the predominant pattern of infiltration, as previously described [10]. The mean number (±SD) of total intratumoral CD3+ T-lymphocytes was 116.6 (±84.3), minimum 26 and maximum 364. The c-kit immunoexpression was mainly cytoplasmic (92.5%, *n* = 74), with a few cases having simultaneous cytoplasmic and membranous labelling (7.5%, *n* = 6; Figure 1). In intratumoral area the labelled cells were mostly epithelial; however immunoreactivity in malignant myoepithelial cells and sarcoma cells of carcinosarcomas was observed also (Figure 2).

**Figure 3:**
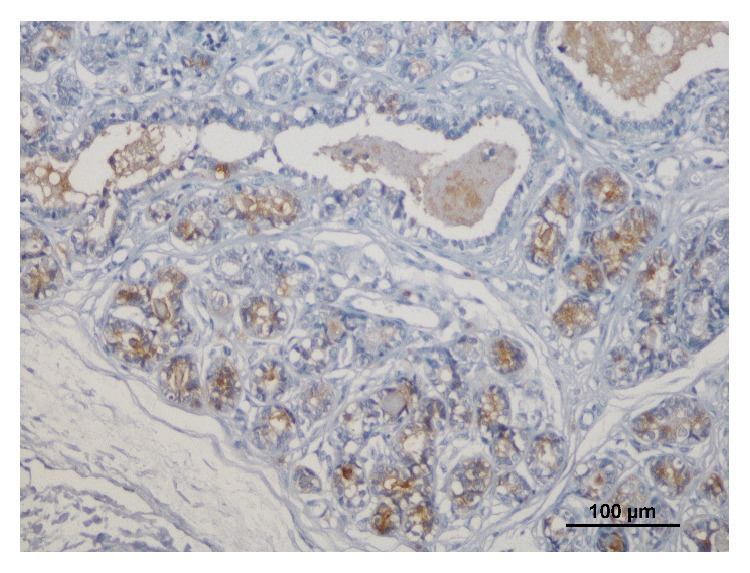
Immunoreactivity for c-kit in adnexal nontumoral mammary gland; bar = 100 *μ*m.

In adnexal nontumoral mammary gland c-kit expression was observed in alveolar and ductal mammary epithelium in smaller proportions relative to the tumor (Figure 3).

For immunolabelling extension, most tumors showed a diffuse immunolabelling (60%, *n* = 48), whereas tumors with intermediate (28.75%, *n* = 23) and focal labelling patterns (11.25%, *n* = 9) were less frequent. For labelling intensity, there was a relatively homogeneous distribution between strong (45%, *n* = 36) and moderate labelling (37.5%, *n* = 30), whereas tumors with weak intensity (17.5%, *n* = 14) were less frequent. The VEGF and CD31 immunostaining followed the classic immunoreactive pattern [30]. In brief, VEGF immunostaining was frequently observed in the cytoplasm of tumor cells and was more intense in epithelial neoplastic cells coating ducts or tubulopapillary formations with a spotted pattern. The CD31 immunoexpression was observed in endothelial cells as a subtle outline of microvessels. The mean number (±SD) of total CD31 was 39.12 (±20.7), minimum 8 and maximum 106.

### 3.2. CD3+ T-Lymphocytes, c-Kit, VEGF, and CD31 Correlations

CD3+ T-cells and high c-kit immunoexpression revealed a positive and significant correlation with CD31 (*r* = 0.654, *P* < 0.0001; *r* = 0.365, *P* = 0.003 for CD3 and c-kit, resp.) and VEGF (*r* = 0.503, *P* < 0.0001; *r* = 0.284, *P* = 0.023 for CD3 and c-kit, resp.). A positive correlation between CD3+ T-lymphocytes and c-kit was also observed (*r* = 0.263, *P* = 0.039).

### 3.3. CD3+ T-Lymphocytes, c-Kit, CD31, and VEGF Expression Associations

**Figure 4:**
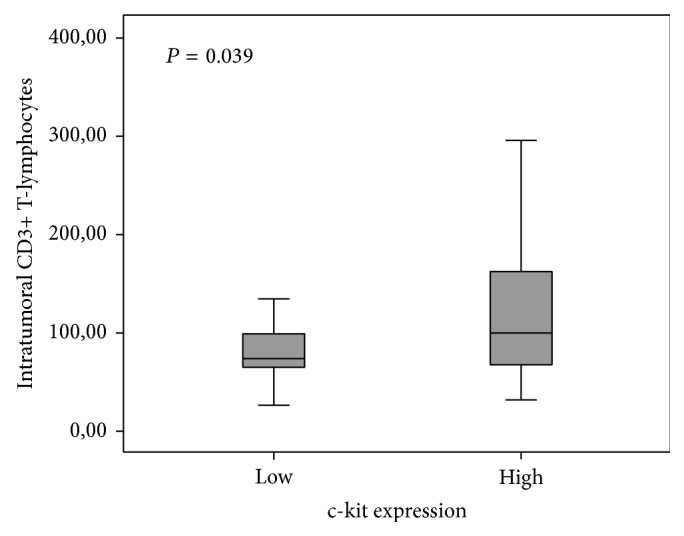
Association of CD3+ T-lymphocytes and c-kit in malignant canine mammary tumors.

A statistically significant association between CD3+ T-lymphocytes and c-kit was observed (*P* = 0.039). Tumors with high c-kit expression showed higher counts of CD3+ T-cells (Figure 4).

**Figure 5:**
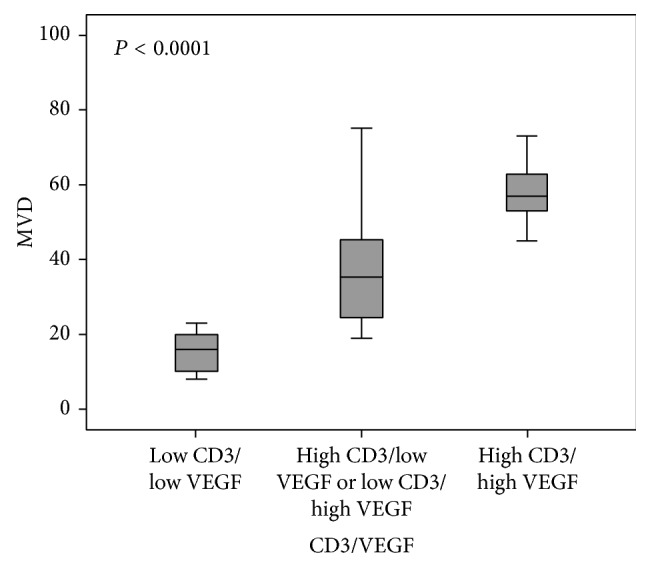
Association of MVD and CD3/VEGF groups in malignant canine mammary tumors.

**Figure 6:**
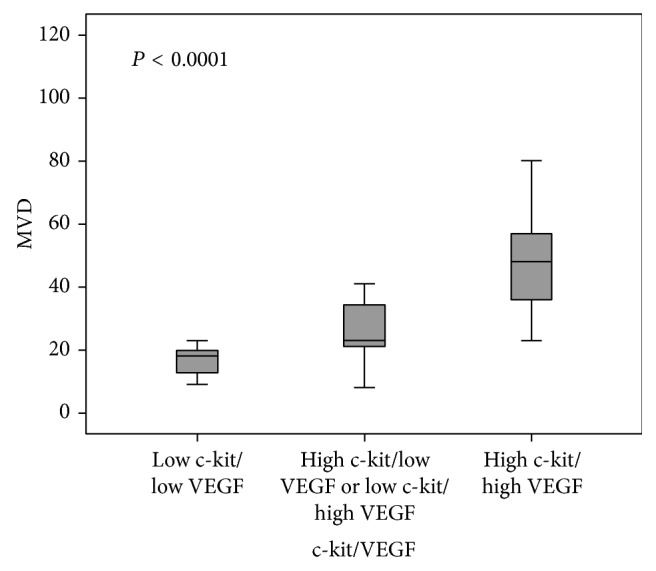
Association of MVD and c-kit/VEGF groups in malignant canine mammary tumors.

The MVD of high CD3/VEGF tumors was significantly more elevated (*P* < 0.0001; Figure 5). A similar association was observed for high c-kit/VEGF tumors (*P* < 0.0001; Figure 6).

### 3.4. Relationship of CD3/c-Kit, CD3/VEGF, and c-Kit/VEGF with Clinicopathological Variables of Tumor Aggressiveness

In this study high CD3/c-kit, high CD3/VEGF, and high c-kit/VEGF tumors were statistically associated with elevated grade of malignancy (*P* < 0.0001 for CD3/c-kit, CD3/VEGF, and c-kit/VEGF), presence of neoplastic intravascular emboli (*P* < 0.0001 for CD3/c-kit and CD3/VEGF; *P* = 0.002 for c-kit/VEGF), and presence of lymph node metastasis (*P* < 0.0001 for CD3/c-kit, CD3/VEGF, and c-kit/VEGF). More information is provided in Table 1.

### 3.5. Comparison between CD3/c-Kit, CD3/VEGF, c-Kit/VEGF, and OS

**Figure 7:**
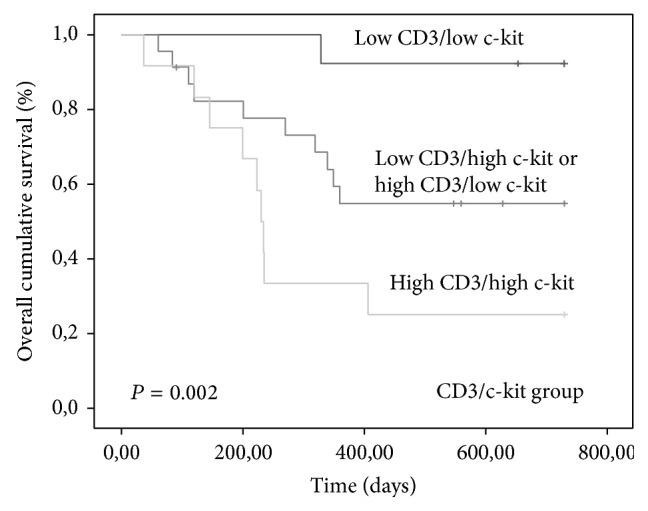
Kaplan-Meier overall survival curves comparing CD3/c-kit group categories in 80 dogs with malignant mammary tumors.

**Figure 8:**
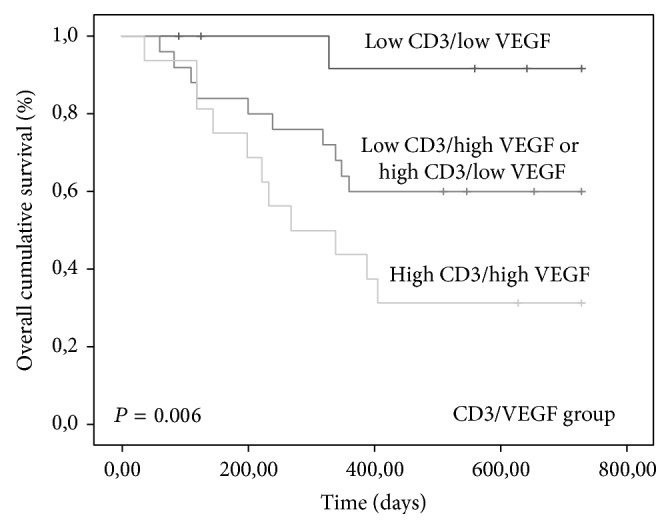
Kaplan-Meier overall survival curves comparing CD3/VEGF group categories in 80 dogs with malignant mammary tumors.

**Figure 9:**
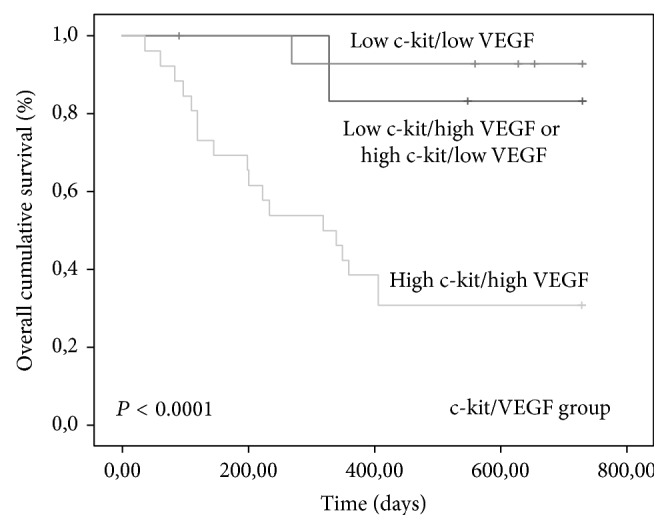
Kaplan-Meier overall survival curves comparing c-kit/VEGF group categories in 80 dogs with malignant mammary tumors.

Tumors with high expression of CD3/c-kit (*P* = 0.002), high CD3/VEGF (*P* = 0.006), and high c-kit/VEGF (*P* < 0.0001) were associated with shorter OS time (Figures 7, 8, and 9).

The group of tumors with high c-kit/VEGF retained their significance by multivariate analysis arising as independent predictor of poor prognosis (Table 2).

## 4. Discussion

During the course of multistep mammary tumorigenesis, tumor cells acquire functional capabilities, via distinct mechanisms, in order to survive and disseminate [34, 35]. One of the most prominent mechanisms involves the inflammatory state of premalignant and malignant mammary lesions coordinated by cells of the immune system, including T-lymphocytes [1, 2]. These immune cells, by deregulation of key signaling pathways, contribute to human and dog mammary tumors autonomy and ability to sustain proliferative and angiogenic switch [2, 36]. The cross-pathways that happen in tumor microenvironment are carried in large part by growth factors that bind cell-surface receptors, typically containing intracellular tyrosine kinase domains, like c-kit [16, 22, 25, 37]. Reports in both species describe T-lymphocytes and c-kit overexpression in mammary tumours and support a role for them in tumor progression [8, 10, 22, 25].

The results of the present study demonstrate a positive correlation of CD3+ T-cells and high c-kit expression with VEGF and CD31. Overall survival study revealed that high c-kit/VEGF group retained the significant relationship with OS by multivariate analysis arising as an independent predictor of poor prognosis. Angiogenesis is crucial in the growth and spread of malignant mammary tumors [38, 39]. The findings of our work are in accordance with recent literature in human breast cancer [39] and in CMT [2, 10, 11] suggesting that CD3+ T-lymphocyte cytokines in mammary tumor sites may stimulate angiogenesis through the induction of proangiogenic factors as VEGF that contribute to new blood vessel formation from preexisting ones and are implicated in mammary tumor aggressiveness and shorter OS times. Our results support also the possibility of common signaling pathways between c-kit and VEGF that have a key role in tumor angiogenic switch, malignancy, and poor prognosis. In dogs there are only a few studies accessing the expression of c-kit in mammary tumorigenesis [25–27]. The lack of other studies in CMT and the controversy of results in human breast cancer studies [22–24] preclude more adequate comparisons of our results. However in small cell lung cancer was demonstrated that the activation of c-kit by stem cells factor (SCF) leads to a predominantly HIF-1-alpha-mediated enhancement of VEGF expression and that inhibition of c-kit signalling with imatinib could result in inhibition of tumor angiogenesis [40].

Our data showed also a significant association and a positive correlation between CD3+ T-lymphocytes and c-kit expression. Furthermore concurrent overexpression of these markers was statistically associated with variables of tumor aggressiveness and shorter OS. In several human tumors, including breast cancer, the SCF that triggers the c-kit signaling pathways has been described as possibly being involved in differentiation, migration, survival, and maturation of T-cells and other inflammatory cells into tumor sites, by a complex relation between mast cells, tumor cells, and T-lymphocytes in the tumor microenvironment [15, 16]. In humans, the upregulation of c-kit by dendritic cells via, induce the activation of PI3 kinase signaling that block IL-12 and promote IL-6 serum production, which in turn supports an immune twisting toward Th2 and Th17 subsets and away from Th1 responses. The Th2 and Th17 cytokines induce T-cell tolerance which is a deleterious immune condition in a variety of diseases, including cancer [15, 17, 18]. All of these immune functions attributed to c-kit disclose the complex relation between c-kit/SCF axis, tumor cells, and T-cells in the tumor microenvironment that could result in chronic inflammation with immune balance from activation to tolerance and may be implicated in mammary tumorigenesis. Therefore, the c-kit/SCF pathways not only are important for the remodeling of tumor microenvironment but also could be a very important target for tumor immunological therapy.

## 5. Conclusion

Results of this study strongly suggest that T-lymphocytes may share common signaling pathways with c-kit and VEGF in CMT progression and may contribute to increased angiogenesis, tumor aggressiveness, and poor prognosis.

## Figures and Tables

**Table 1:** Relationship of CD3/c-kit, CD3/VEGF, and c-kit/VEGF groups with clinicopathological variables of tumor aggressiveness.

		Variables of tumor aggressiveness								
Molecular markers		HGM			Neoplastic intravascular emboli			Lymph node metastasis		
		Low (I/II)	High (III)	*P*	Absent	Present	*P*	Absent	Present	*P*
CD3/c-kit	Low CD3/low c-kit	18	4	<0.0001	20	2	<0.0001	18	4	<0.0001
	Low CD3/high c-kit or high CD3/low c-kit	19	16		27	8		24	11	
	High CD3/high c-kit	3	20		8	15		7	16	

CD3/VEGF	Low CD3/low VEGF	17	3	<0.0001	18	2	<0.0001	16	4	<0.0001
	Low CD3/high VEGF or high CD3/low VEGF	21	14		28	7		26	9	
	High CD3/high VEGF	2	23		9	16		7	18	

c-kit/VEGF	Low c-kit/low VEGF	10	2	<0.0001	12	0	0.002	11	1	<0.0001
	Low c-kit/high VEGF or high c-kit/low VEGF	20	6		22	4		21	5	
	High c-kit/high VEGF	10	32		21	21		17	25	

*n*, number of samples; *P*, statistical significance; NS, not significant.

**Table 2:** Association between considered molecular markers groups and overall survival.

Molecular markers		*n*	Overall survival Univariate (mean values)	*P*	Overall survival Multivariate^*∗*^ (hazard ratio)	*P*
CD3/c-kit	Low CD3/low c-kit	22	699.154	0.002	—	NS
	Low CD3/high c-kit or high CD3/low c-kit	35	500.809			
	High CD3/high c-kit	23	335.083			

CD3/VEGF	Low CD3/low VEGF	20	696.583	0.006	—	NS
	Low CD3/high VEGF or high CD3/low VEGF	35	524.480			
	High CD3/high VEGF	25	383.500			

c-kit/VEGF	Low c-kit/low VEGF	12	663.167	<0.0001	6.102	0.010
	Low c-kit/high VEGF or high c-kit/low VEGF	26	697.143			
	High c-kit/high VEGF	42	371.462			

*n*, number of samples; *P*, statistical significance; NS, not significant; ^*∗*^Cox proportional hazard model: forward stepwise method-likelihood ratio.
